# Supplementary material for: Global, Regional, and National Burden of Protein–Energy Malnutrition: A Systematic Analysis for the Global Burden of Disease Study
Source: Nutrients. 2022 Jun 22;14(13):2592. doi: 10.3390/nu14132592 (PMC9268010; doi:10.3390/nu14132592)
Supplement: Supplementary file 1 [file nutrients-14-02592-s001.zip › nutrients-1750239-supplementary.pdf]

Supplementary Information

Figure S1. Average annual percentage change (AAPC) of the (A) years of life lost (YLL) and (B) years lived with disability (YLD) rate for protein–energy malnutrition from 1990 to 2019. AAPC was obtained representing the average percent increase or decrease in PEM rates per year over each specified period of time to summarize and compare these trends over the entire time period.

Figure S2. The global disease burden of protein–energy malnutrition for both genders in 204 countries and territories. (A) The age-standardized death rate (ASDR) of protein–energy malnutrition in 2019. (B) The estimated annual percentage change (EAPC) in ASDR of protein–energy malnutrition from 1990 to 2019.

Figure S3. The global disease burden of protein–energy malnutrition for both genders in 204 countries and territories. (A) The age-standardized DALY rate of protein–energy malnutrition in 2019. (B) The estimated annual percentage change (EAPC) in the age-standardized DALY rate of protein–energy malnutrition from 1990 to 2019.

Figure S4. The years of life lost (YLL) and years lived with disability (YLD) rates of protein–energy malnutrition among gender and age. (A) YLL rate. (B) YLD rate.

Figure S5. Age-standardized (A) death rates, and (B) disability-adjusted life year for protein–energy malnutrition for 21 GBD regions and by Socio-Demographic Index, 1990–2019. There was clearly an ASR downtrend of the prediction curve when the SDI went up. Expected values based on Socio-Demographic Index and disease rates in all locations are shown as the black line.

Figure S6. Age-standardized (A) prevalence, (B) death, and (C) DALY rates for PEM for 195 countries and territories by Socio-demographic Index, 1990–2019. There was a clearly ASR downtrend of the prediction curve when the SDI went up. Expected values based on Socio-demographic Index and disease rates in all locations are shown as the black line.

Figure S7. The association between Human Development Index (HDI) and estimated annual percentage change (EAPC) in age-standardized prevalence rate (ASPR), age-standardized death rate (ASDR), and age-standardized disability-adjusted life years of protein–energy malnutrition. HDI (2019) was negatively associated with EAPC in (A) ASPR ($\rho = -0.53$, P < 0.001), (B) APSR ($\rho = -0.42$, P < 0.001), and (C) age-standardized DALY ($\rho = -0.64$, P < 0.001).

Figure S8. The proportion of the five age groups for protein–energy malnutrition death cases between 1990 and 2019 globally, and in High, High-middle, Middle, Low-middle, and Low SDI quintiles. The populations were divided into five age groups: under 5, 5-14 years, 15-49 years, 50-69 years, and 70+ years. SDI values can be used to judge the degree of economic development of a country or region.

Figure S9. The proportion of the five age groups for protein–energy malnutrition DALY cases between 1990 and 2019 globally, and in High, High-middle, Middle, Low-middle, and Low SDI quintiles. The populations were divided into five age groups: under 5, 5-14 years, 15-49 years, 50-69 years, and 70+ years. SDI values can be used to judge the degree of economic development of a country or region.

Figure S10. JointPoint regression analysis of the age-standardized prevalence rates for PEM from 1990 to 2019. (A) The age-standardized prevalence rate in High SDI quintiles; (B) Age-standardized prevalence rate in High-middle SDI quintiles; (C) Age-standardized prevalence rate in Middle SDI quintiles; (D) Age-standardized prevalence rate in Low-middle SDI quintiles; (E) Age-standardized prevalence rate in Low SDI quintiles. The prevalence of High SDI, High-middle SDI, and Middle SDI quintiles increased from 1990 to 2011, and the Low-middle SDI and Low SDI decreased at the

same time. The prevalence of all SDI groups went through a U-shaped curve from 2011 to 2019.

Figure S11. JointPoint regression analysis of the age-standardized death rates for PEM from 1990 to 2019. (A) The age-standardized death rate in High SDI quintiles; (B) Age-standardized death rate in High-middle SDI quintiles; (C) Age-standardized death rate in Middle SDI quintiles; (D) Age-standardized death rate in Low-middle SDI quintiles; (E) Age-standardized death rate in Low SDI quintiles. As for the JointPoint of deaths, High-middle SDI, Middle SDI, and Low SDI decreased from 1990 to 2019 and the High SDI and Low-middle SDI decreased from 2000 to 2019.

Figure S12. JointPoint regression analysis of the age-standardized DALY rates for PEM from 1990 to 2019. (A) Age-standardized DALY rate in High SDI quintiles; (B) Age-standardized DALY rate in High-middle SDI quintiles; (C) Age-standardized DALY rate in Middle SDI quintiles; (D) Age-standardized DALY rate in Low-middle SDI quintiles; (E) Age-standardized DALY rate in Low SDI quintiles. The JointPoint of all SDI groups except the High SDI group decreased over time.

Table S1. The death cases and age-standardized death of PEM in 1990 and 2019, and its temporal trends from 1990 to 2019.

Table S2. The DALY cases and age-standardized DALY of PEM in 1990 and 2019, and its temporal trends from 1990 to 2019.

Table S1. The death cases and age-standardized death of PEM in 1990 and 2019, and its temporal trends from 1990 to 2019.

	1990		2019		1990-2019
	Death cases	ASR per 100,000	Death cases	ASR per 100,000	EAPC
	No. (95% UI)	No. (95% UI)	No. (95% UI)	No. (95% UI)	No. (95% CI)
Global	656313.6 (495905.4-877702.4)	11.9 (9.1-15.5)	212242.1 (185402.8-246217)	3 (2.6-3.5)	-5.15 (-5.5--4.8)
Social-demographic index					
Low SDI	235171.1 (180589.6-313513.1)	36.7 (29.4-46.6)	94539.2 (75150.1-119848.7)	9.8 (8.1-12)	-4.74 (-4.9--4.58)
Low-middle SDI	296837.5 (196325.9-426549.6)	24.5 (16.3-34.2)	44468.2 (37729.8-52975.1)	3.3 (2.9-3.9)	-7.87 (-8.78--6.95)
Middle SDI	96102.4 (82811.5-111317.2)	7.9 (7-8.8)	45104.2 (40443-49013.4)	2.5 (2.2-2.7)	-3.93 (-4.11--3.75)
High-middle SDI	21159.9 (18701.8-24247.1)	2.2 (2-2.5)	13680.8 (11895.1-14893.5)	0.8 (0.7-0.9)	-3.85 (-4.17--3.53)
High SDI	6707.6 (5774.5-7320.7)	0.7 (0.6-0.8)	14291.9 (11310.4-15824.9)	0.6 (0.5-0.7)	-0.76 (-0.96--0.55)
Region					
Central Asia	368 (321.7-421)	0.5 (0.4-0.5)	131.1 (110.8-149.9)	0.2 (0.2-0.2)	-3.43 (-3.71--3.14)
Central Europe	128.6 (116.6-145.4)	0.1 (0.1-0.1)	333.6 (233.9-382.3)	0.2 (0.1-0.2)	1.06 (0.5-1.63)
Eastern Europe	471.8 (367.9-536.6)	0.2 (0.2-0.2)	342.8 (231.2-399.2)	0.1 (0.1-0.1)	-2.92 (-3.46--2.38)
Australasia	53.9 (44.2-66.3)	0.3 (0.2-0.3)	147.9 (118.3-171)	0.3 (0.2-0.3)	-0.47 (-0.85--0.08)
High-income Asia Pacific	578.9 (520.6-727.3)	0.3 (0.3-0.4)	1518.6 (1159.1-1733.9)	0.3 (0.2-0.3)	-1.29 (-1.77--0.79)
High-income North America	2579.6 (2194.5-2763.1)	0.7 (0.6-0.8)	6012.1 (5023.1-6518.5)	0.8 (0.7-0.9)	0.17 (-0.19-0.54)
Southern Latin America	1524.5 (1407.8-1633.2)	3.4 (3.1-3.6)	1909.1 (1665.1-2095)	2.3 (2-2.5)	-1.8 (-2.42--1.17)
Western Europe	3090 (2580.9-3473.7)	0.6 (0.5-0.7)	7179.3 (5420.7-8344.1)	0.6 (0.4-0.7)	0.01 (-0.05-0.07)
Andean Latin America	6225.2 (5302.5-7322.6)	18.2 (16-20.5)	2597 (2115-3118.7)	4.7 (3.9-5.7)	-5.26 (-5.51--5)
Caribbean	4546.8 (3493.1-6019.2)	12.2 (9.7-15.8)	1741.8 (1331.2-2295.8)	3.9 (2.9-5.2)	-3.63 (-4.06--3.19)
Central Latin America	19927.9 (17045.3-21456)	19 (15.4-20.3)	11074.5 (9433-12628.4)	5 (4.2-5.7)	-5.05 (-5.2--4.9)
Tropical Latin America	15725.1 (14174.7-17657.6)	12.7 (11.5-13.9)	7946.5 (6884.3-8615.3)	3.7 (3.2-4)	-4.48 (-4.67--4.3)
North Africa and Middle East	15915.9 (10559.8-29411.6)	4.1 (2.9-7)	4940.7 (3919.4-6594.8)	1.2 (1-1.5)	-4.53 (-4.67--4.39)
South Asia	282829.2 (169738.5-415458.3)	22.9 (13.6-33.3)	25109.4 (18221.6-33268.2)	1.7 (1.2-2.2)	-8.77 (-8.92--8.61)
East Asia	42000.8 (36143.9-48328.3)	5.1 (4.5-5.7)	13579 (11603.2-15450.5)	1.1 (0.9-1.2)	-8.82 (-11.27--6.31)
Oceania	300.2 (241-375.7)	8.8 (7.5-10.3)	302.6 (224.1-421)	4.7 (3.7-6.3)	-2.04 (-2.17--1.91)
Southeast Asia	43849.9 (29464.1-57134.8)	14.8 (10.6-17.7)	24989.2 (21975.1-27500.3)	5.5 (4.8-6.1)	-3.07 (-3.34--2.81)
Central Sub-Saharan Africa	27355.3 (18607.9-39308.2)	42.1 (32.4-54.2)	10497.4 (7374.1-14800.4)	12.8 (9.1-17.8)	-4.01 (-4.41--3.6)
Eastern Sub-Saharan Africa	127411.8 (99224.3-165143.7)	63.8 (51.7-79.4)	49774.1 (40552.2-62294.5)	17.6 (14.6-20.7)	-4.72 (-4.89--4.54)
Southern Sub-Saharan Africa	8498.1 (6882.2-10410.8)	15.8 (13.6-18.4)	5535.4 (4424.8-6866.3)	8.3 (6.9-10.2)	-1.71 (-1.94--1.47)
Western Sub-Saharan Africa	52932.2 (40387.3-67848.1)	21.1 (17.4-25.4)	36579.9 (27465.1-48179.9)	8.5 (6.8-10.7)	-3.48 (-3.75--3.21)

Table S2. The DALY cases and age-standardized DALY of PEM in 1990 and 2019, and its temporal trends from 1990 to 2019.

	1990		2019		1990-2019
	DALY cases	ASR per 100,000	DALY cases	ASR per 100,000	EAPC
	No. (95% UI)	No. (95% UI)	No. (95% UI)	No. (95% UI)	No. (95% CI)
Global	52743908.1 (40250363.1-70481551.6)	855.3 (654.3-1138.2)	15256524.2 (12565113.6-18327802.9)	218.3 (179.5-262.8)	-5.03 (-5.27--4.79)
Social-demographic index					
Low SDI	19211755.5 (14708633.7-25949125.4)	2212.5 (1717.4-2942.8)	7617058.5 (5999962.7-9645847.9)	524.6 (422.1-652.2)	-5.13 (-5.27--4.99)
Low-middle SDI	24444271.9 (16525828.1-34962690.2)	1552.9 (1055.4-2209.1)	3729158.4 (3056888.7-4507283)	222.8 (183.8-268.2)	-7.28 (-7.81--6.74)
Middle SDI	7090465 (6089965.2-8474681.5)	388.9 (337.5-455.7)	2491723.5 (2013108.7-3038168.2)	118.6 (96.8-142.9)	-4.15 (-4.32--3.98)
High-middle SDI	1635820.8 (1399234.3-1921583.3)	156.4 (134.2-183.9)	882525.8 (643211-1161046.6)	64 (46.1-85.1)	-3.43 (-3.71--3.16)
High SDI	336117 (254323.4-440884.8)	38.7 (29-51.2)	526299.8 (390895.7-690306.8)	40 (28.2-55)	-0.19 (-0.28--0.09)
Region					
Central Asia	50004.9 (40531.5-60232.1)	58.1 (47.6-69.9)	30296.1 (21060.7-41052)	32.8 (23-44.3)	-2.42 (-2.55--2.29)
Central Europe	26249.6 (18784.2-35914.2)	24.4 (17.7-32.6)	29992.4 (21271.8-41670.4)	26.4 (18-37.4)	-0.13 (-0.28-0.03)
Eastern Europe	65878.3 (48795.4-88163.2)	32.1 (23.7-43)	54555.2 (37648.4-76582.8)	28.9 (19.6-40.8)	-1.14 (-1.37--0.91)
Australasia	3337.5 (2370.6-4631.5)	16 (11.3-22.4)	6488.4 (4695.2-8851.4)	18.3 (12.6-25.8)	0.31 (0.14-0.48)
High-income Asia Pacific	35577.7 (27006.6-47122.5)	20.2 (15.3-26.7)	50490.1 (38928-65083)	20.2 (14.5-27.6)	-0.56 (-0.85--0.27)
High-income North America	89245 (68712.8-117733.4)	28.4 (21.2-38.5)	163453.3 (129364.4-205374.2)	33.5 (25-44.2)	0.1 (-0.09-0.29)
Southern Latin America	85632.7 (77751.3-94710.1)	172.5 (157-190.5)	41891.3 (36969.7-47027.5)	57.3 (50.2-64.9)	-4.23 (-4.55--3.91)
Western Europe	207939.5 (144923.8-288449.3)	48.4 (32.8-68.8)	331120.5 (234716.4-443062.7)	58 (38.7-81.7)	0.44 (0.3-0.57)
Andean Latin America	425581.6 (352256.6-519112.2)	880.3 (740.5-1053.4)	83976.5 (64967.3-105113.3)	139.4 (108.3-173.4)	-6.92 (-7.18--6.66)
Caribbean	356913.3 (267955.9-486329.7)	873 (661.7-1184.2)	106350.2 (75786.6-151585.7)	254.3 (178-366.2)	-3.87 (-4.32--3.42)
Central Latin America	1090198.5 (969608.6-1200048.6)	640.6 (556.9-696.4)	375493.6 (318561.5-446480.9)	162.6 (137.4-193.9)	-5.06 (-5.19--4.93)
Tropical Latin America	1127287.7 (1000554.2-1286087)	707.5 (629.9-802.3)	245192.4 (216192-274543.2)	123.1 (107.6-140)	-6.53 (-6.82--6.23)
North Africa and Middle East	1425848 (975818.5-2566601)	288 (201.8-510.2)	506176.9 (390162.6-681963.3)	87.8 (68.5-117.3)	-4.45 (-4.63--4.26)
South Asia	24562743.7 (15674936.9-35552701.2)	1596.8 (1022.8-2302.7)	3645460.9 (2809838.2-4616537)	217.6 (167.9-275.5)	-6.92 (-7.15--6.68)
East Asia	3176825.4 (2655376.8-3727244.4)	277.3 (234.5-322.9)	816395.1 (572420.2-1111906.8)	54.6 (39.7-73.9)	-8.99 (-11.16--6.77)
Oceania	22700.7 (17755.3-28922.4)	314.3 (259.7-385.4)	23551.6 (17555-32009.5)	171.5 (132.9-226.4)	-1.92 (-2.12--1.73)
Southeast Asia	2857700.6 (1961018.6-3943041.3)	593.7 (420.1-783.1)	1064141.8 (872005.6-1266714.3)	185.7 (154.3-218.8)	-3.9 (-4.15--3.64)
Central Sub-Saharan Africa	2211668.3 (1479913.5-3251527.6)	2334 (1644.3-3278.3)	738212.1 (520971.8-1039644.4)	492.9 (360.2-672.9)	-5.17 (-5.68--4.66)
Eastern Sub-Saharan Africa	9878708 (7575392-12980236.3)	3290.9 (2586.9-4235)	3500613.4 (2756804.3-4470743.5)	716.6 (585.7-886)	-5.4 (-5.57--5.22)
Southern Sub-Saharan Africa	656313 (518633.8-825158.2)	1001 (808.4-1236.6)	377762.1 (288766.7-484396.5)	488.5 (377.9-621.1)	-1.62 (-2.23--1.01)
Western Sub-Saharan Africa	4387553.9 (3318636.7-5678503)	1331.8 (1030.1-1702.4)	3064910.3 (2281373.2-4008485.3)	479.3 (367.4-615)	-3.92 (-4.21--3.63)

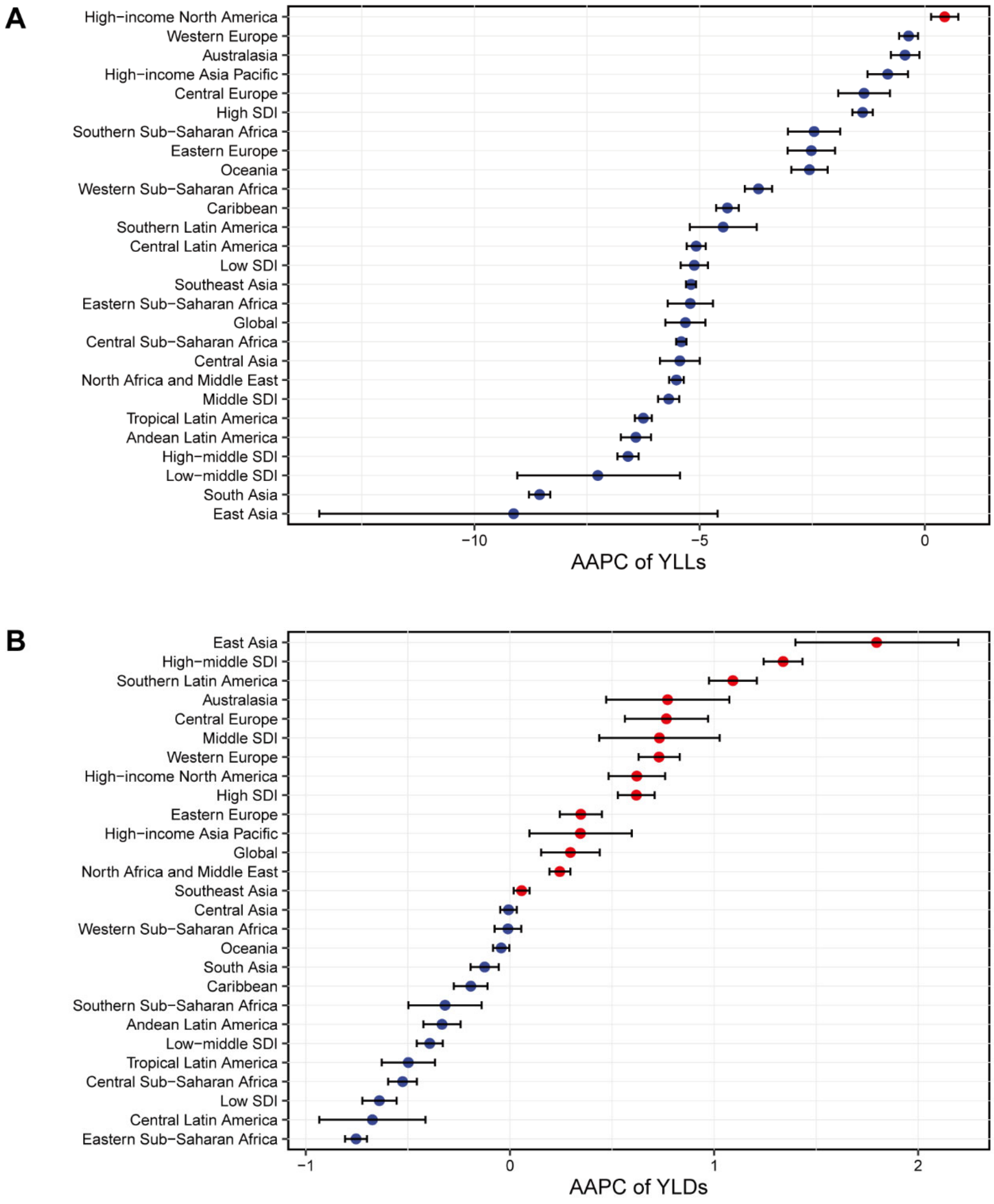
A
High−income North America
Western Europe
Australasia
High−income Asia Pacific
Central Europe
High SDI
Southern Sub−Saharan Africa
Eastern Europe
Oceania
Western Sub−Saharan Africa
Caribbean
Southern Latin America
Central Latin America
Low SDI
Southeast Asia
Eastern Sub−Saharan Africa
Global
Central Sub−Saharan Africa
Central Asia
North Africa and Middle East
Middle SDI
Tropical Latin America
Andean Latin America
High−middle SDI
Low−middle SDI
South Asia
East Asia
−10
−5
0
AAPC of YLLs
B
East Asia
High−middle SDI
Southern Latin America
Australasia
Central Europe
Middle SDI
Western Europe
High−income North America
High SDI
Eastern Europe
High−income Asia Pacific
Global
North Africa and Middle East
Southeast Asia
Central Asia
Western Sub−Saharan Africa
Oceania
South Asia
Caribbean
Southern Sub−Saharan Africa
Andean Latin America
Low−middle SDI
Tropical Latin America
Central Sub−Saharan Africa
Low SDI
Central Latin America
Eastern Sub−Saharan Africa
−1
0
1
2
AAPC of YLDs

Figure S1

A

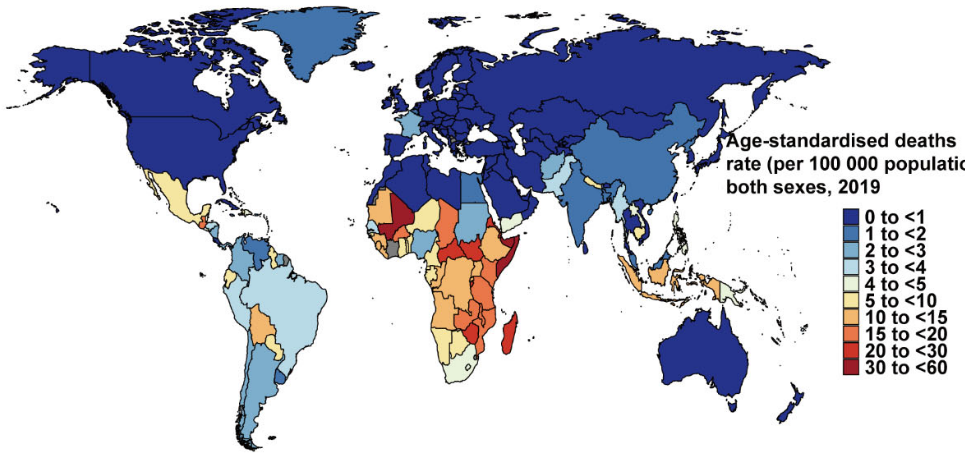

Age-standardised deaths rate (per 100 000 populatic both sexes, 2019
0 to <1
1 to <2
2 to <3
3 to <4
4 to <5
5 to <10
10 to <15
15 to <20
20 to <30
30 to <60

B

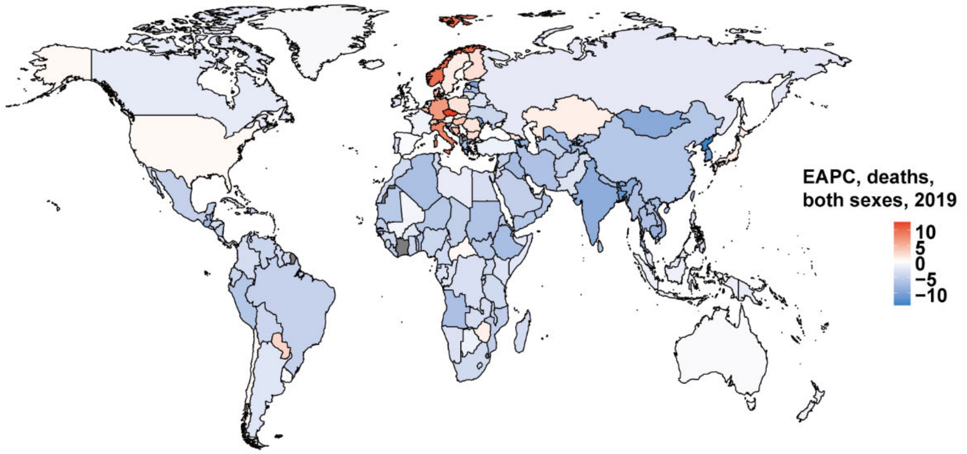

EAPC, deaths, both sexes, 2019
10
5
0
−5
−10

Figure S2

A

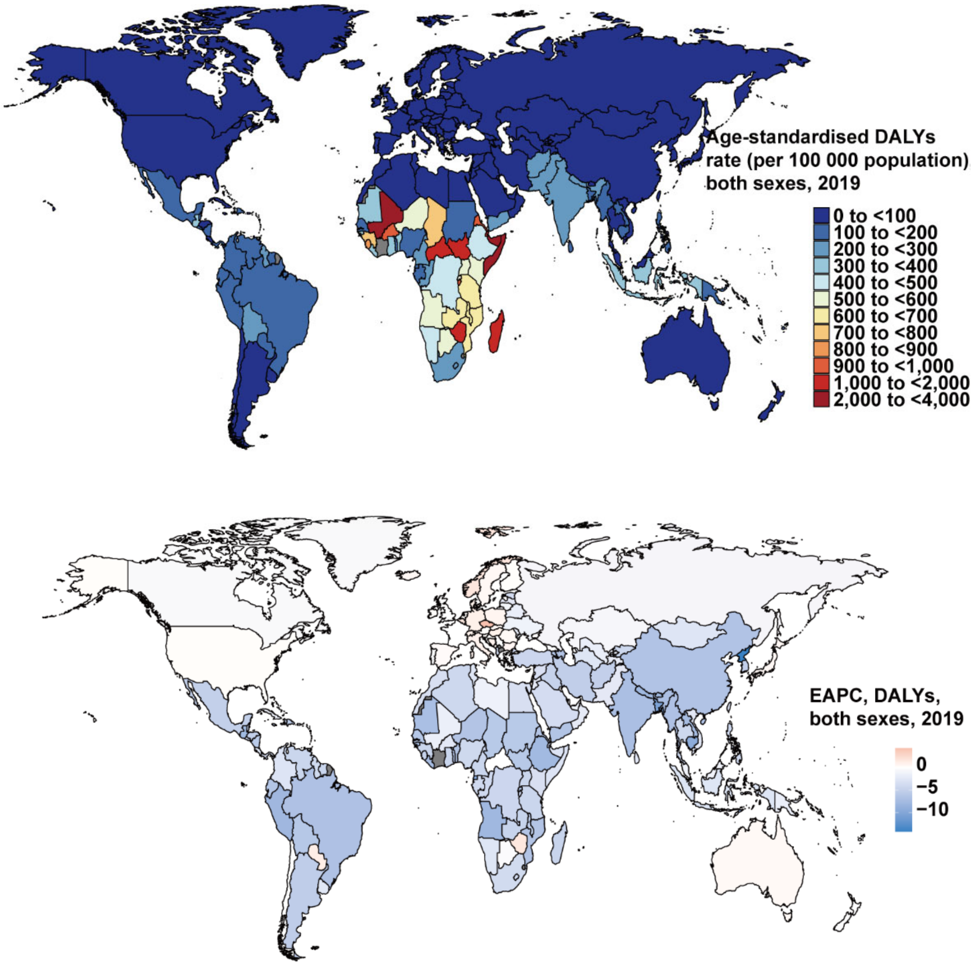
Age-standardised DALYs rate (per 100 000 population) both sexes, 2019
0 to <100
100 to <200
200 to <300
300 to <400
400 to <500
500 to <600
600 to <700
700 to <800
800 to <900
900 to <1,000
1,000 to <2,000
2,000 to <4,000
B
EAPC, DALYs, both sexes, 2019
0
−5
−10

Figure S3

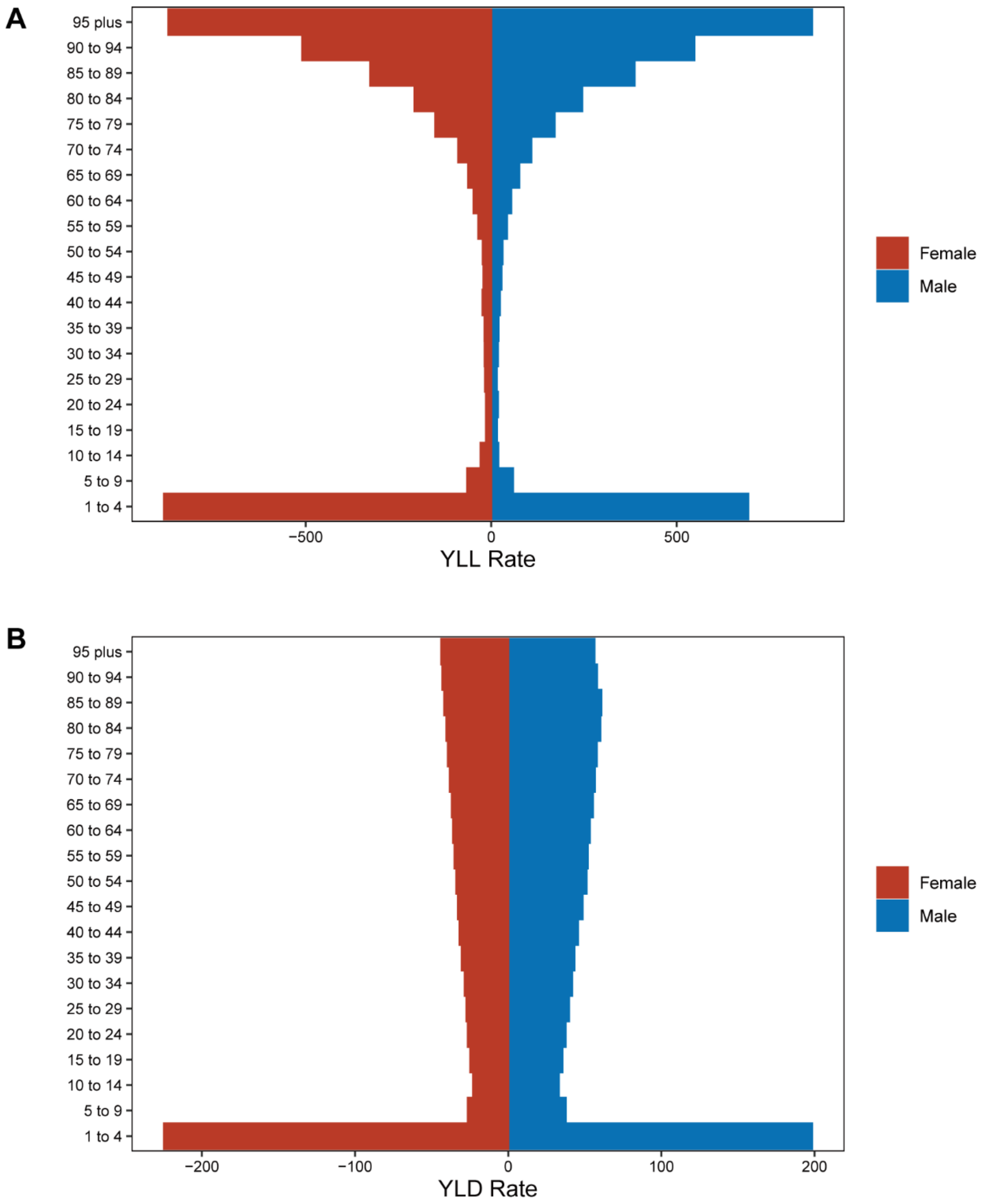
A
95 plus
90 to 94
85 to 89
80 to 84
75 to 79
70 to 74
65 to 69
60 to 64
55 to 59
50 to 54
45 to 49
40 to 44
35 to 39
30 to 34
25 to 29
20 to 24
15 to 19
10 to 14
5 to 9
1 to 4
−500
0
500
YLL Rate
Female
Male
B
95 plus
90 to 94
85 to 89
80 to 84
75 to 79
70 to 74
65 to 69
60 to 64
55 to 59
50 to 54
45 to 49
40 to 44
35 to 39
30 to 34
25 to 29
20 to 24
15 to 19
10 to 14
5 to 9
1 to 4
−200
−100
0
100
200
YLD Rate
Female
Male

Figure S4

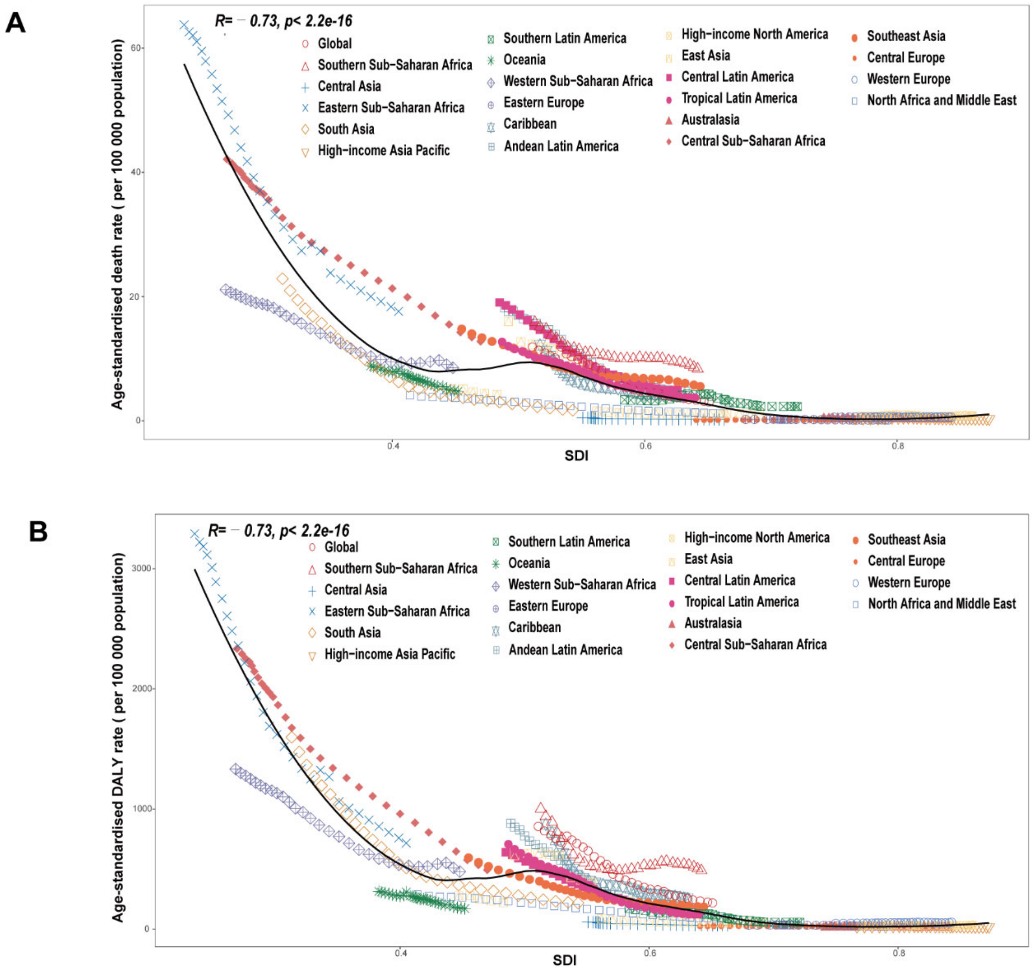
A
R= − 0.73, p< 2.2e-16
Global
Southern Sub−Saharan Africa
Central Asia
Eastern Sub−Saharan Africa
South Asia
High−income Asia Pacific
Southern Latin America
Oceania
Western Sub−Saharan Africa
Eastern Europe
Caribbean
Andean Latin America
High−income North America
East Asia
Central Latin America
Tropical Latin America
Australasia
Central Sub−Saharan Africa
Southeast Asia
Central Europe
Western Europe
North Africa and Middle East
Age-standardised death rate (per 100 000 population)
SDI
B
R= − 0.73, p< 2.2e-16
Age-standardised DALY rate (per 100 000 population)
SDI

Figure S5

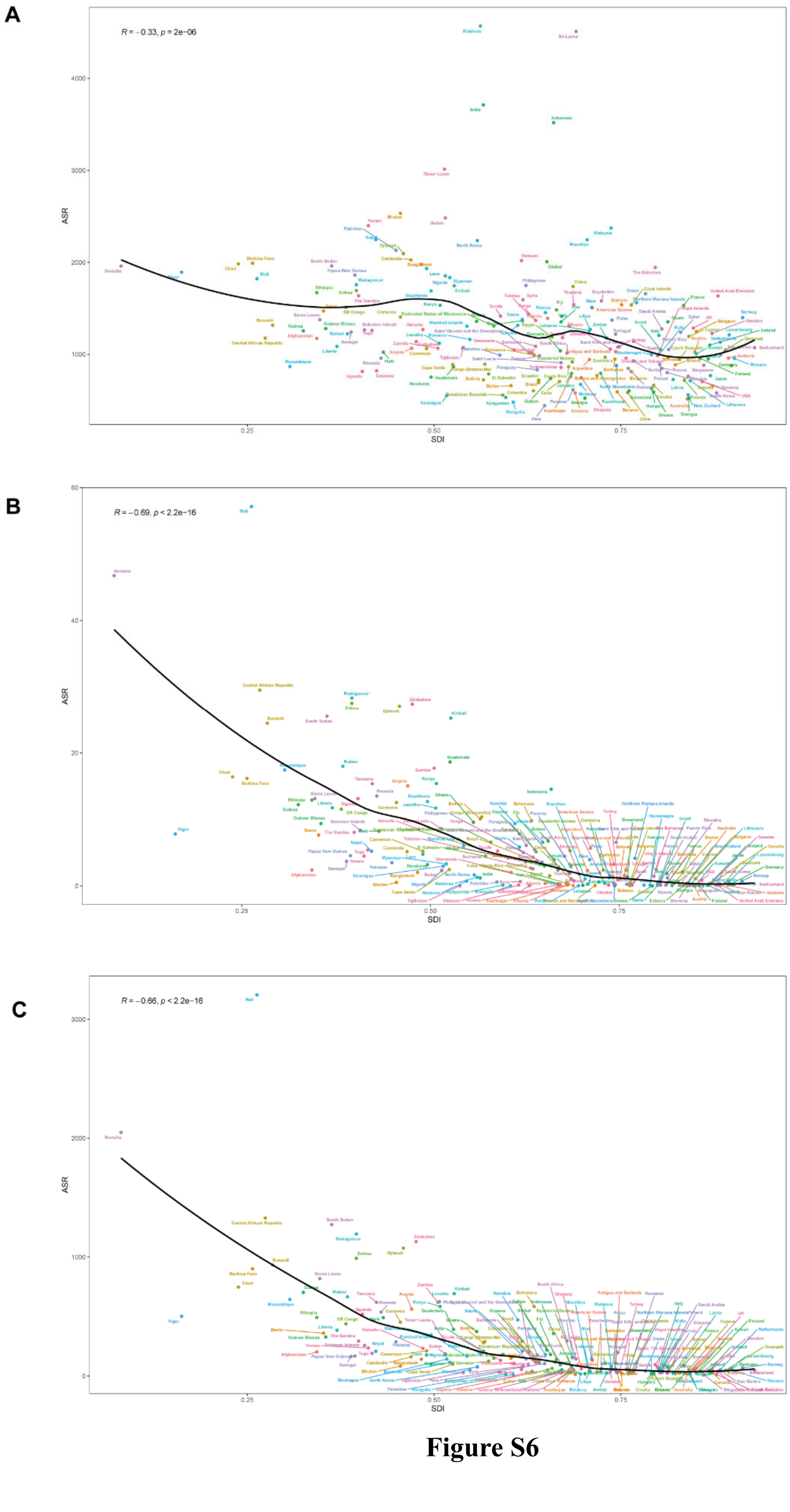
A
R = −0.33, p = 2e−06
ASR
SDI
B
R = −0.69, p < 2.2e−16
ASR
SDI
C
R = −0.66, p < 2.2e−16
ASR
SDI

Figure S6

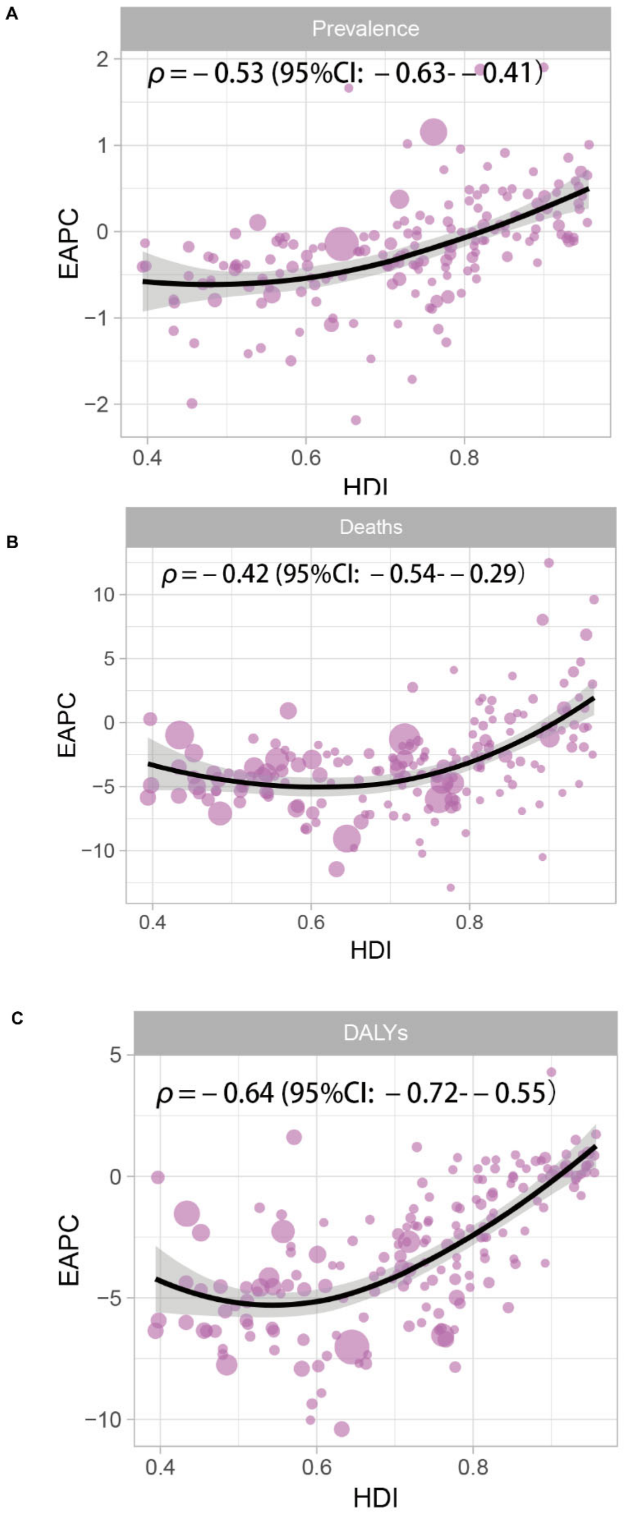
A
Prevalence
ρ = − 0.53 (95%CI: − 0.63- − 0.41)
EAPC
HDI
B
Deaths
ρ = − 0.42 (95%CI: − 0.54- − 0.29)
EAPC
HDI
C
DALYs
ρ = − 0.64 (95%CI: − 0.72- − 0.55)
EAPC
HDI

Figure S7

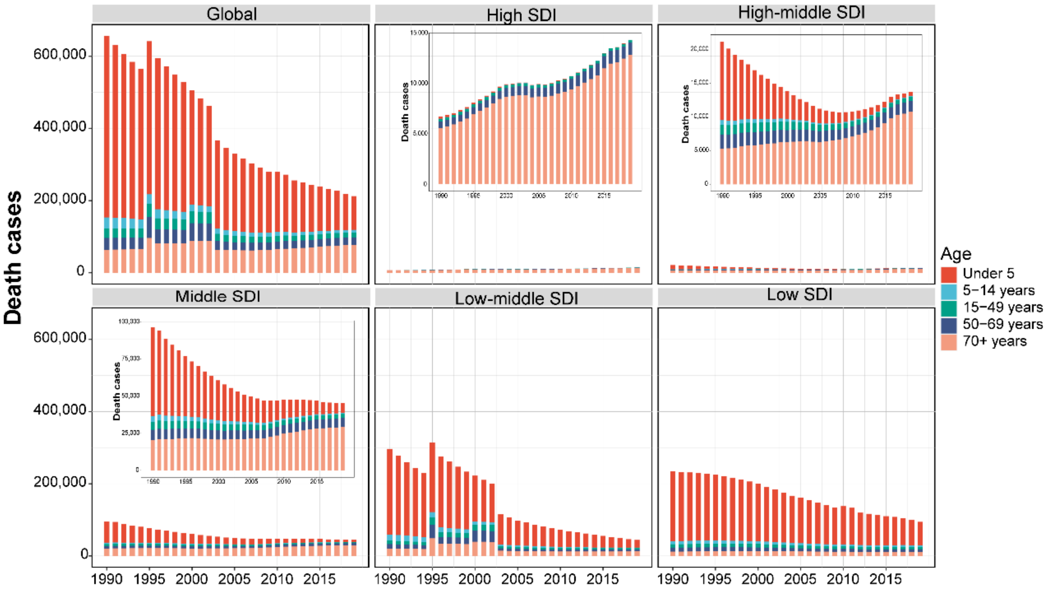

Global
High SDI
High-middle SDI
Middle SDI
Low-middle SDI
Low SDI
Death cases
0
200,000
400,000
600,000
1990
1995
2000
2005
2010
2015
Age
Under 5
5−14 years
15−49 years
50−69 years
70+ years

Figure S8

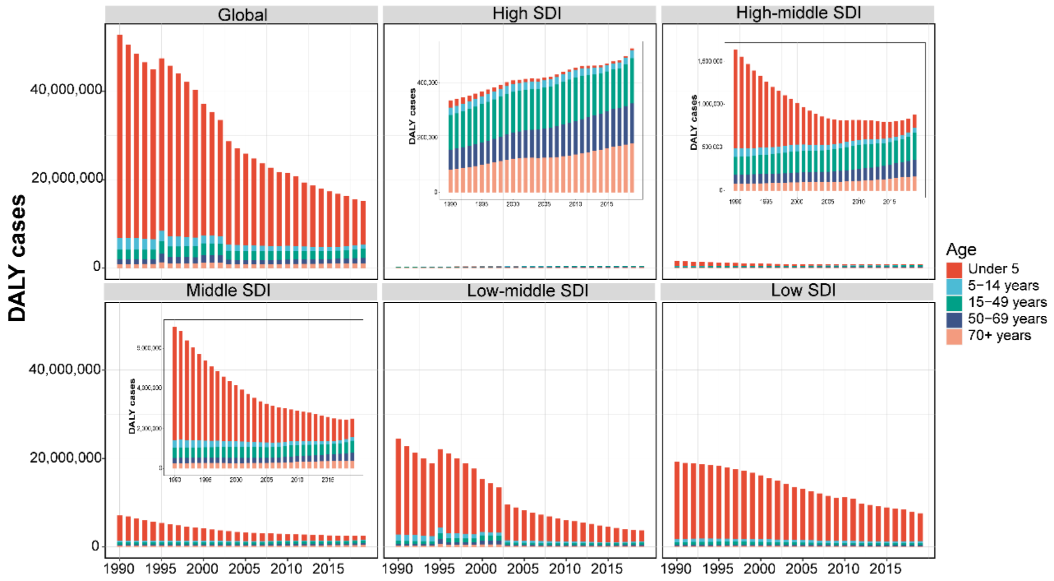
Global
High SDI
High-middle SDI
Middle SDI
Low-middle SDI
Low SDI
DALY cases
40,000,000
20,000,000
0
1990
1995
2000
2005
2010
2015
Age
Under 5
5−14 years
15−49 years
50−69 years
70+ years

Figure S9

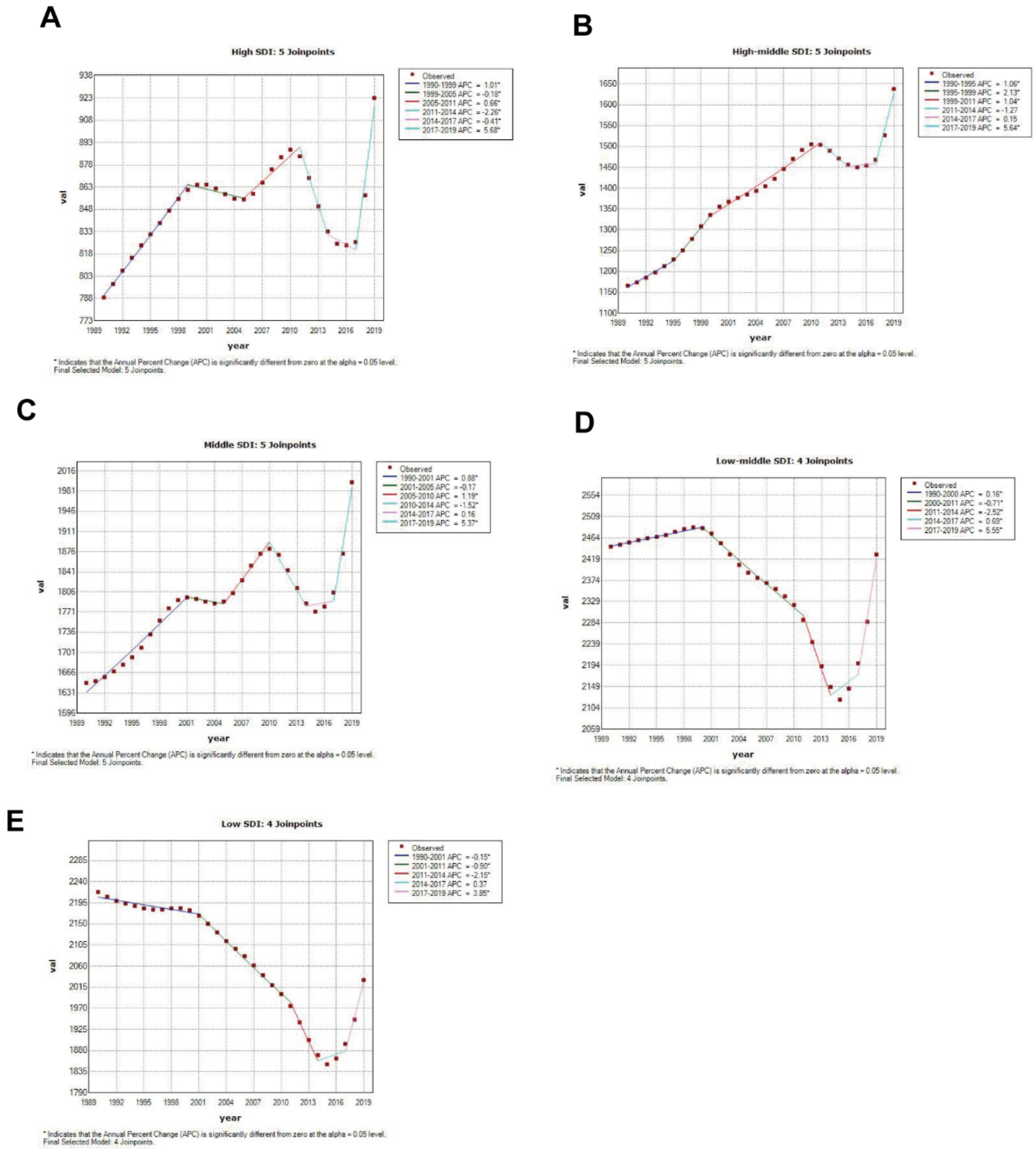
A
High SDI: 5 Joinpoints
Observed
1990-1999 APC = 1.01*
1999-2005 APC = -0.18*
2005-2011 APC = 0.66*
2011-2014 APC = -2.26*
2014-2017 APC = -0.41*
2017-2019 APC = 5.68*
val
year
* Indicates that the Annual Percent Change (APC) is significantly different from zero at the alpha = 0.05 level.
Final Selected Model: 5 Joinpoints.
B
High-middle SDI: 5 Joinpoints
Observed
1990-1995 APC = 1.06*
1995-1999 APC = 2.13*
1999-2011 APC = 1.04*
2011-2014 APC = -1.27
2014-2017 APC = 0.15
2017-2019 APC = 5.64*
val
year
* Indicates that the Annual Percent Change (APC) is significantly different from zero at the alpha = 0.05 level.
Final Selected Model: 5 Joinpoints.
C
Middle SDI: 5 Joinpoints
Observed
1990-2001 APC = 0.88*
2001-2005 APC = -0.17
2005-2010 APC = 1.19*
2010-2014 APC = -1.52*
2014-2017 APC = 0.16
2017-2019 APC = 5.37*
val
year
* Indicates that the Annual Percent Change (APC) is significantly different from zero at the alpha = 0.05 level.
Final Selected Model: 5 Joinpoints.
D
Low-middle SDI: 4 Joinpoints
Observed
1990-2000 APC = 0.16*
2000-2011 APC = -0.71*
2011-2014 APC = -2.52*
2014-2017 APC = 0.69*
2017-2019 APC = 5.55*
val
year
* Indicates that the Annual Percent Change (APC) is significantly different from zero at the alpha = 0.05 level.
Final Selected Model: 4 Joinpoints.
E
Low SDI: 4 Joinpoints
Observed
1990-2001 APC = -0.15*
2001-2011 APC = -0.90*
2011-2014 APC = -2.15*
2014-2017 APC = 0.37
2017-2019 APC = 3.85*
val
year
* Indicates that the Annual Percent Change (APC) is significantly different from zero at the alpha = 0.05 level.
Final Selected Model: 4 Joinpoints.

Figure S10

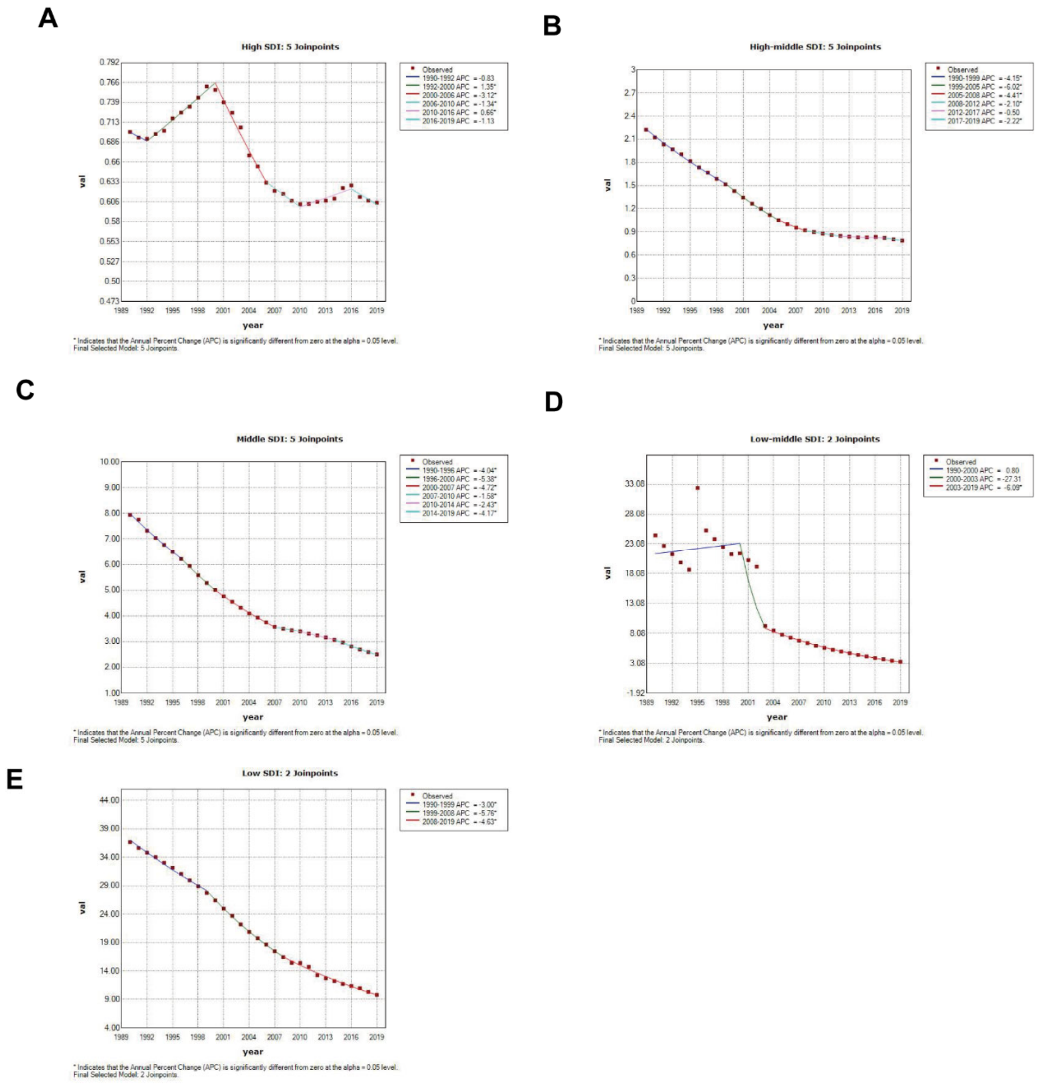
A
High SDI: 5 Joinpoints
Observed
1990-1992 APC = -0.83
1992-2000 APC = 1.35*
2000-2006 APC = -3.12*
2006-2010 APC = -1.34*
2010-2016 APC = 0.66*
2016-2019 APC = -1.13
val
year
* Indicates that the Annual Percent Change (APC) is significantly different from zero at the alpha = 0.05 level.
Final Selected Model: 5 Joinpoints.
B
High-middle SDI: 5 Joinpoints
Observed
1990-1999 APC = -4.15*
1999-2005 APC = -6.02*
2005-2008 APC = -4.41*
2008-2012 APC = -2.10*
2012-2017 APC = -0.50
2017-2019 APC = -2.22*
val
year
* Indicates that the Annual Percent Change (APC) is significantly different from zero at the alpha = 0.05 level.
Final Selected Model: 5 Joinpoints.
C
Middle SDI: 5 Joinpoints
Observed
1990-1996 APC = -4.04*
1996-2000 APC = -5.38*
2000-2007 APC = -4.72*
2007-2010 APC = -1.58*
2010-2014 APC = -2.43*
2014-2019 APC = -4.17*
val
year
* Indicates that the Annual Percent Change (APC) is significantly different from zero at the alpha = 0.05 level.
Final Selected Model: 5 Joinpoints.
D
Low-middle SDI: 2 Joinpoints
Observed
1990-2000 APC = 0.80
2000-2003 APC = -27.31
2003-2019 APC = -6.09*
val
year
* Indicates that the Annual Percent Change (APC) is significantly different from zero at the alpha = 0.05 level.
Final Selected Model: 2 Joinpoints.
E
Low SDI: 2 Joinpoints
Observed
1990-1999 APC = -3.00*
1999-2008 APC = -5.76*
2008-2019 APC = -4.63*
val
year
* Indicates that the Annual Percent Change (APC) is significantly different from zero at the alpha = 0.05 level.
Final Selected Model: 2 Joinpoints.

Figure S11

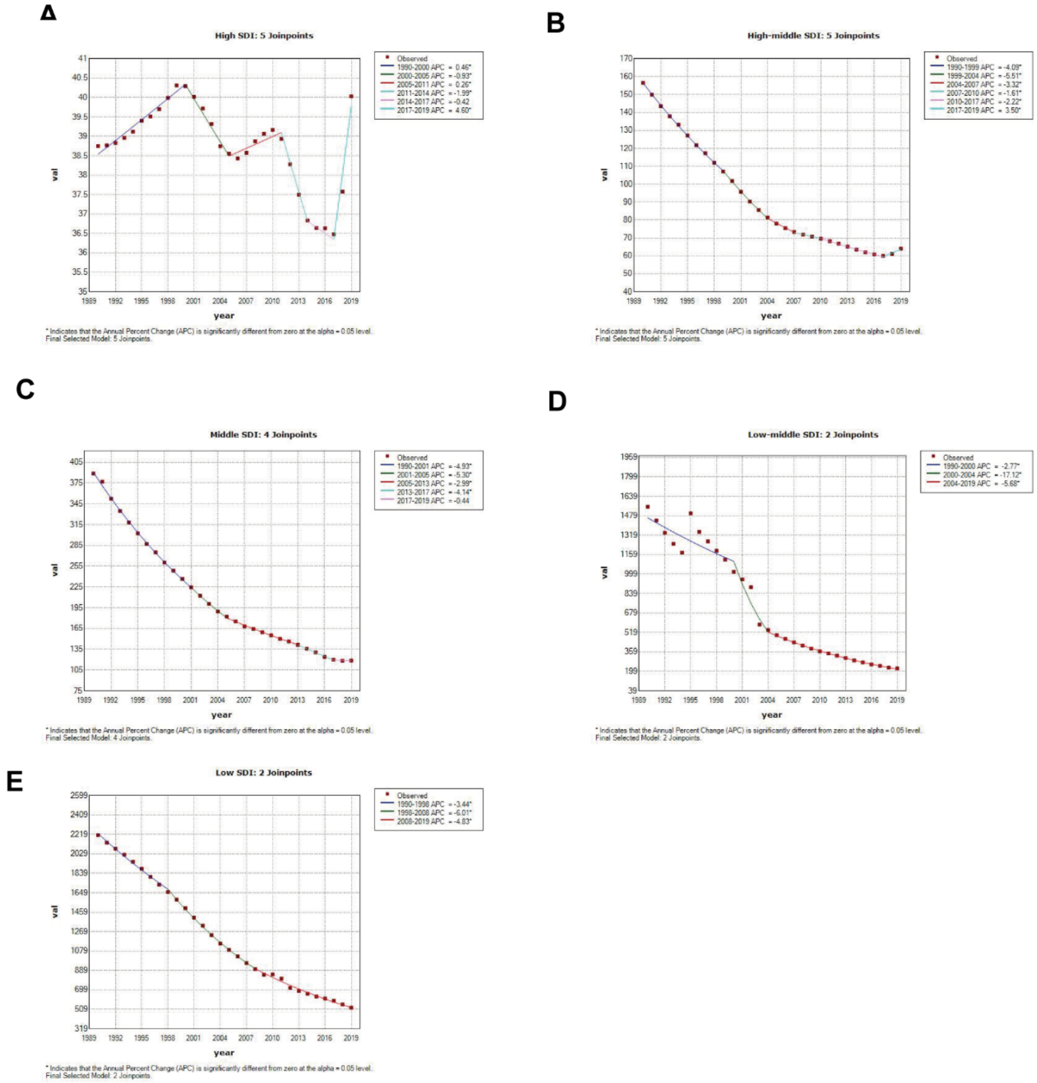
A
High SDI: 5 Joinpoints
Observed
1990-2000 APC = 0.46*
2000-2005 APC = -0.93*
2005-2011 APC = 0.26*
2011-2014 APC = -1.99*
2014-2017 APC = -0.42
2017-2019 APC = 4.60*
val
year
* Indicates that the Annual Percent Change (APC) is significantly different from zero at the alpha = 0.05 level.
Final Selected Model: 5 Joinpoints.
B
High-middle SDI: 5 Joinpoints
Observed
1990-1999 APC = -4.09*
1999-2004 APC = -5.51*
2004-2007 APC = -3.32*
2007-2010 APC = -1.61*
2010-2017 APC = -2.22*
2017-2019 APC = 3.50*
val
year
* Indicates that the Annual Percent Change (APC) is significantly different from zero at the alpha = 0.05 level.
Final Selected Model: 5 Joinpoints.
C
Middle SDI: 4 Joinpoints
Observed
1990-2001 APC = -4.93*
2001-2005 APC = -5.30*
2005-2013 APC = -2.99*
2013-2017 APC = -4.14*
2017-2019 APC = -0.44
val
year
* Indicates that the Annual Percent Change (APC) is significantly different from zero at the alpha = 0.05 level.
Final Selected Model: 4 Joinpoints.
D
Low-middle SDI: 2 Joinpoints
Observed
1990-2000 APC = -2.77*
2000-2004 APC = -17.12*
2004-2019 APC = -5.68*
val
year
* Indicates that the Annual Percent Change (APC) is significantly different from zero at the alpha = 0.05 level.
Final Selected Model: 2 Joinpoints.
E
Low SDI: 2 Joinpoints
Observed
1990-1998 APC = -3.44*
1998-2008 APC = -6.01*
2008-2019 APC = -4.83*
val
year
* Indicates that the Annual Percent Change (APC) is significantly different from zero at the alpha = 0.05 level.
Final Selected Model: 2 Joinpoints.

Figure S12
